# *In-vitro* and *in-vivo* evaluations of tocotrienol-rich nanoemulsified system on skin wound healing

**DOI:** 10.1371/journal.pone.0267381

**Published:** 2022-05-25

**Authors:** Wai Ting Chong, Chin Ping Tan, Yoke Kqueen Cheah, Oi Ming Lai

**Affiliations:** 1 Institute of Bioscience, Universiti Putra Malaysia, Serdang, Selangor, Malaysia; 2 Department of Food Technology, Faculty of Food Science and Technology, Universiti Putra Malaysia, Serdang, Selangor, Malaysia; 3 Department of Biomedical Sciences, Faculty of Medicine and Health Sciences, Universiti Putra Malaysia, Selangor, Malaysia; 4 Department of Bioprocess Technology, Faculty Biotechnology and Biomolecular Sciences, Universiti Putra Malaysia, Serdang, Selangor, Malaysia; Central University of Rajasthan, INDIA

## Abstract

Proper wound healing is vital for the survival of higher organisms. Responses to skin injury can lead to complications such as scar formation that can affect the quality of life. In this study, keratinocytes migration (scratch assay) and zebrafish tail regeneration experiments were used to evaluate the wound healing effect of a tocotrienol-based nanoemulsified (NE) system against ascorbic acid and phosphate-buffered saline (PBS) as positive and negative controls, respectively. MTT assay provided a concentration range of 0.35–8.75 μg/ml of nanoemulsion that produced cell viability more than 100%. After 24 hours of treatment, the wound closure of keratinocytes were found to be significantly faster by 73.76%, 63.37% and 35.56%, respectively when treated with 3.50 μg/ml and 1.75 μg/ml of NE compared to the blank. The lethal concentration at 50% (LC_50_ value) obtained from acute and prolonged toxicity was almost similar, which was 4.6 mg/ml and 5.0 mg/ml, respectively. Growth of zebrafish tail regeneration treated with NE at a concentration of 2.5 mg/ml was significantly faster than the untreated zebrafish, which regenerated to 40% on the fifth day, more than 60% on the tenth day of treatment and fully recovered at the twentieth day. In conclusion, these results showed the potential of the tocotrienols-based nanoemulsified system in enhancing wound healing through accelerated wound closure.

## 1. Introduction

Wounds are defined as damaged part of the body which occurred through cut, burn, scratch and/or breakdown of skin. In developed countries, around 2% of the general population suffers from wound problems [1, 2] and it remains a growing health care issue among the high-risk populations (e.g. diabetic, obese, smokers or the aged) [3]. The harm caused by wound problems can lead to impaired and delayed healing of wounds, pain, stress and scarring which can cause complications and affect the quality of life. Therefore, novel and less invasive topical therapeutic applications of wound management are in demand.

Vitamin E refers to a group of fat-soluble vitamins known as tocopherols and tocotrienols. Palm oil (940 mg/kg total tocotrienols), rice bran oil (464 mg/kg total tocotrienols) and annatto (Bixa Orellana L.) (1400–1470 mg/kg total tocotrienols) are considered some of the richest sources of tocotrienols [4, 5]. Annatto is the richest source of tocotrienols with tocopherols: tocotrienols ratio of 0.1:99.9 but a few studies reported that annatto seed causes allergic reactions [6–8]. Natural vitamin E from crude palm oil which is also commonly known as tocotrienol-rich fraction consists of 70% tocotrienols and 30% tocopherols [4].

Numerous *in vitro* studies indicate that tocotrienols exhibit anti cancer, cardioprotective and neuroprotective effects [4] and many of its effects can be linked to its antioxidant function. A study by [9] showed that tocotrienols have better antioxidant potency than tocopherols by a factor of 60 times. The antioxidant potential of tocotrienols is derived from their “chain-breaking” ability to quench peroxyl and alkoxyl radicals generated during lipid peroxidation [4]. The beneficial effect of tocotrienols in dermatology especially in treating melasma and skin inflammation has also been reported [10–13]. Zampieri et al. [14] and Eileen et al. [15] reported on the positive effects of topical vitamin E in surgical wound repair. Although the effect of tocotrienols in dermatology has been shown, to the best of our knowledge, *in vitro* and *in vivo* studies using red palm oil (RPO), which contains high amounts of carotenoids (500-700ppm) and vitamin E (500-1000ppm tocopherols and tocotrienols) in wound healing is limited. The development of topical applications containing natural plant bioactives that promotes wound healing process is desired.

Therefore, the present study was undertaken to evaluate the potential effects of a tocotrienol-based nanoemulsified system on wound healing using *in-vitro* (keratinocytes) and *in-vivo* (zebrafish) models.

## 2. Materials and methods

### 2.1 Materials

Commercially available red palm oil (RPO) was purchased from a local supplier in Malaysia. RPO is non-GMO-certified and contains 664.994±1.946 ppm (0.44:0.56, α-carotene: β-carotene) of carotenoids and 999.485±20.023 ppm of vitamin E, respectively. Polyoxyethylenesorbitan monooleate (Tween 80), sorbitan monoolete (Span 80) and glycerol were obtained from SystermChemAR (Shah Alam, Malaysia). The water used was Ultrapure water from Milli-Q Plus. For high-performance liquid chromatography (HPLC) analysis, isopropanol, hexane, methanol, acetonitrile, ethyl acetate, triethylamine and 1,4-dioxane were purchased from Fisher Scientific (Loughborough, United Kingdom). Ascorbic acid and DPPH (2, 2 diphenyl-1-picryl hydrazyl) were obtained from TCI America (Tokyo, Japan), while ethanol was obtained from Merck (Darmstadt, Germany). Primary epidermal keratinocytes (normal human, adult, PCS- 200–011) and complete growth medium were purchased from American Type Culture Collection (Virginia, ATCC, USA). Tricaine (MS-222) was purchased from Sigma. Dimethyl sulfoxide (DMSO) and 3-(4,5- Dimethylthiazol-2-yl)-2,5-diphenyltetrazolium bromide for dye (MTT) from MP Biomedicals (France) were used in this study.

### 2.2 Determination of carotenoids and vitamin E

To extract the active ingredients in red palm oil (RPO), 1.0 mL of water and internal standard (t*rans* β-apo-8-carotenal for carotenoids analysis and 2,2,5,7,8- pentamethyl-6-chromanol for Vitamin E analysis) were added into centrifuge tube. The solution was vortexed for 1 minute. Then, 1.0 mL of isopropanol was added into the solution and vortexed for 1 minute. 10 mL of hexane then was added into the solution and vortexed for 2 minutes. The solution was centrifuged at 2500 rpm for 15 minutes at 4°C. After centrifuge, upper organic layer was drawn out and transferred to a glass vial. Lastly, the transferred organic solution was dried with nitrogen gas. Oil extraction was conducted and dried samples were reconstituted with solvent diluents for HPLC analysis.

The carotenoids (Model LC-20AD, Shimadzu, Kyoto, Japan) and vitamin E (Model Prominence i, Shimadzu, Kyoto, Japan) in RPO were evaluated by HPLC following a method described in [16, 17], respectively with minor modifications. For carotenoids analysis, isocratic mobile phase using a solvent mixture of methanol:acetonitrile:ethyl acetate (70:15:15, v/v/v, 0.1% v/v triethylamine) at 0.8mL/min on a ODS Hypersil column (5 μm) (250 x 4.6 mm) (Thermo Scientific, Waltham, USA) resulted in an analysis time of 25 minutes, at 445nm and 450nm wavelength (UV-Vis detector). While for vitamin E, isocratic mobile phase using a solvent mixture of n-hexane: dioxane: isopropanol (97.5:2.0:0.5, v/v/v) at 1 mL/min on a Phenomenex Luna (Torrance, USA) 5 μm Silica C18 (2) 100 liquid chromatography column (250, 4.6 mm) resulted in an analysis time of 30 minutes, at 295nm (fluorescence detector excitation) and 325nm wavelength (fluorescence detector emission).

### 2.3 Preparation of tocotrienol-rich nanoemulsion system

Oil phase consisted of RPO and surfactant blend (Tween 80: Span 80 at 6:4 wt) while water phase consisted of Ultrapure water and glycerol. The nanoemulsions prepared in this system were of 5 wt% surfactant-blend, 10 wt% glycerol, 20 wt% RPO and 65 wt% water. The surfactant blend ratio of 6:4 used in this study was based on the optimized results that were conducted using a central composite design (CCD) coupled to RSM [18]. The RSM analysis showed that the experimental data could be fitted into a second-order polynomial model with coefficients of multiple determination (R2) of 0.9115. The oil phase was added to the water phase and premixed using high shear (Silverson L4R, Buckinghamshire, UK) at 6000 rpm for 10 minutes and pH adjusted to 7 for tonicity. The premixed mixtures then were passed through high pressure homogenizer (Panda 2 K, Niro Soavi, Deutschland, Lubeck, Germany).

### 2.4 Droplet size and polydispersity index analysis

Droplet size and polydispersity index (PDI) of nanoemulsion was measured using Zetasizer Nano ZS (Worcestershire, U.K.) at 25°C. PDI is a measure of the droplet size distribution. It shows the percentage of droplets in distinct size classes.

### 2.5 Antioxidant content

DPPH (2, 2 diphenyl-1-picryl hydrazyl) assay method developed by [19] was used to determine the antioxidant activity. To produce the DPPH solution, 2.3 mg of DPPH was added to 100 mL ethanol. An aliquot of 0.1 g of sample was added with 3.9 mL of DPPH solution in a test tube. The test tube was vortexed and left in the dark for 30 minutes. The absorbance was read at 515 nm using a spectrophotometer. Ascorbic acid is used as the standard and 95% ethanol is used as blank [20]. According to [21] the percentage of antioxidant activity is calculated as below:

$$Antioxidant\;Activity\;\left(\text{\%}\right)=100-\left[\frac{\left(Abs\;s\text{ample}-Abs\;blank\right)100}{Abs\;control}\right]$$ (1)

### 2.6 Thermodynamic stability studies

Thermodynamic stability studies were carried out to evaluate the meta-stability of nanoemulsions. Nanoemulsions went through centrifugation, heating cooling cycle and freeze-thaw cycle [22] as stated below:

Centrifugation: 10 mL of nanoemulsion sample was filled into a 15 mL bottle. It was centrifuged at 3000 rpm for 30 minutes. The observations of nanoemulsions were recorded.

Heating cooling cycle: The nanoemulsion sample was kept at 4°C for 24 hours in a chiller and the nanoemulsion sample was transferred into an oven at 40°C f or another 24 hours. There were six cycles of thermal cycling. The phase separation was observed and recorded.

Freeze-thaw cycle: The nanoemulsion sample was kept at -21°C for 24 hours and it was stored at 25°C for another 24 hours. There are three cycles of this stability test. The phase separation and droplets size were observed and recorded.

### 2.7 Cell culture and MTT assay

The keratinocyte cell line was cultured in a complete growth medium that contained 0.4% bovine pituitary extract, rh-transforming growth factor, L-glutamine, hydrocortisone hemi-succinate, rh-insulin, epinephrine, Apo-transferrin and antibiotics (penicillin) were maintained at 37°C, 5% CO _2_ humidified incubator. The media were changed after three days. The cells were harvested using trypsin-EDTA when the keratinocytes reached confluence. MTT assay was used to evaluate the cell viability by using a method by Schuh et al. [23]. The keratinocytes (5.0 x 10^3^ cells) were seeded in a 96-well cell culture plate. Nanoemulsion was diluted to 0.35, 3.50, 8.75, 17.50 & 35.00 μg/mL with PBS and added into each well. Blank contained only phosphate-buffered saline (PBS) was used as negative control and ascorbic acid at 4.44 μg/mL used as positive control. After 24 hours of treatments and incubation, 20 μL of MTT dye (5 mg/ml) was added into each well and incubated for another 3–4 hours. Media was removed and 100 μL of DMSO was added into each well. Absorbance was measured at 590 nm using a micro-plate reader (Biolog, USA).

### 2.8 Scratch assay with keratinocytes cell line

The scratch assay was evaluated by a method described in [24] with minor modification. The keratinocytes (1.5 x 10^4^ cells) were seeded in 96-well cell culture plate and incubated overnight. Straight scratch was made using 10 μL pipette tip. Cell debris was washed out with PBS. Nanoemulsion was added into each well at the concentration of 1.75 μg/mL and 3.50 μg/mL (the concentrations were selected based on the results in MTT assay). The positive and negative controls used were the same as the controls used in MTT assay. The images of the cellular gap were captured using Nikon Eclipse TS100 inverted microscope. The cellular gap of the cells was observed after 24 hours and the area of the wound was measured using Image J software. The migration of keratinocytes was expressed as the percentage of wound closure (%) and calculated with the following equation:

$$Wound\;Closure\;\left(\text{\%}\right)=[\left(A0h-A24h)/A0h\right]\;\times \;100\text{\%}$$ (2)

Where, A_0h_ is the area of wound after the scratch was made; A_24h_ is the area of wound after 24 hours of treatment.

### 2.9 Zebrafish maintenance

Keeping and raising of zebrafish (*Danio rerio* F. Hamilton, 1822) broodstocks is performed under approval of Institutional Animal Care and Use Committee (IACUC), Universiti Putra Malaysia, under file number UPM/IACUC/AUP-R062/2020. Adult zebrafish (wild-type shortfin, aged 6–9 months) were maintained in the sampling tank (5 fish/L) with unchanged content for the period of testing at room temperature (25–28°C). Adult zebrafish were fed twice daily in the morning and afternoon with specially formulated zebrafish feeds. The endpoints for wound healing tests are death/ infection/ skin lesion observed on the zebrafish. Any abnormalities that could arise to adult zebrafish include behavioural changes such as restlessness, poor appetite, less responsive, less mobility, and reduction in weight. These observations will trigger the decision to remove an animal from the experiment, or to terminate the experiment. The animals will be euthanised using ice water at 0°C. Carcass will be frozen and keep in the -20°C freezer in Biotech 2 Building, Faculty of Biotechnology and Science Biomolecules, Universiti Putra Malaysia. Then, the National Institute of Occupational Safety and Health (NIOSH) will collect to dispose.

### 2.10 Acute and prolonged toxicity

The zebrafish embryotoxicity assay was carried out based on Organization for Economic Cooperation and Development (OECD) guideline for fish acute toxicity test (OECD TG 203) and fish prolonged toxicity test: 14-day study (OECD 204). Briefly, zebrafish (5 fish/L) were exposed to samples by submerged testing. Fish in the untreated tank was considered as the blank, negative control. The cumulative survival of fish was observed and determined every 24 h from 0–48 hours of post exposure (hpe) and 0–14 days of post exposure (dpe) for acute and prolonged toxicity tests, respectively. At the end of the exposure period, data was obtained by comparing the groups treated with different concentration of nanoemulsion, ascorbic acid and the untreated (control). The lethal concentration at 50% (LC_50_) of nanoemulsion was measured based on the survival rate at 48 hpe for acute toxicity and 14 dpe for prolonged toxicity test.

For Acute toxicity, solutions of 1% (v/v) nanoemulsion were prepared by dissolving 10 mL of the received stock solution into 1 L of treated water. Solutions in the range between 0.25% and 0.5% (v/v) were prepared by dissolving 2.5 ml and 5.0 ml of nanoemulsions, respectively into 1 L of aquarium water. For prolonged toxicity test, the solutions of 0.125%, 0.25% and 0.5% (v/v) used by dissolving 1.25 ml, 2.5 ml and 5 ml of nanoemulsion into 1L of aquarium water. Preparation for 0.1% (v/v) of ascorbic acid was done by dissolving 1 mL of received stock solution (1mg/ml) into 1 L of aquarium water.

### 2.11 Wound healing evaluation

Wound healing test using adult zebrafish (>6 months old) is based on the method described by [25] with some modifications. After undergone acclimatization for two weeks, healthy fish were selected regardless of the gender. The fishes were anaesthetized with tricaine for approximately 20 seconds and the tail was amputated. The length of the fish was then measured immediately under a sterilized operation. After fish had regained recovery in the recovery tank, it was transferred into the sampling tanks. Preparation for 1.0 mg/ml of ascorbic acid and 2.5mg/ml were done by dissolving 1g of ascorbic acid and 2.5g of nanoemulsions into 1L of aquarium water. Measurement of tail regeneration was recorded every five days [after day post amputation (dpa)] interval until 20 days. The images of the zebrafish tail were captures using digital camera (Nikon, Tokyo, Japan) and analyzed using Image J software.

Percentage of tail regeneration (%) is calculated according to the following equation:

$$\begin{array}{l} \text{Tail}\;\text{regeneration}\;\left(\%\right)\;=\frac{\text{Length}\;\text{of}\;\text{tail}\;\text{regenerate}*}{\text{Length}\;\text{of}\;\text{detached}\;\text{tail}\;\left(0\;\text{dpa}\right)}\times 100\\ {}*\left[\text{Tail}\;\text{length}\;\left(\text{before}\;\text{cut}\right)\;-\;\text{Tail}\;\text{length}\;\left(\text{after}\;\text{treatment}\right)\right] \end{array}$$ (3)

### 2.12 Statistical analysis

SPSS was used for data analysis. Analysis of variance (ANOVA), paired-samples T test and Turkey’s post hoc test were applied to determine the significant differences among values under studied conditions.

## 3. Results and discussions

### 3.1 Characterization of the tocotrienol-rich nanoemulsion system

The RPO used in the preparation of the tocotrienol-rich nanoemulsified system contained 664.994±1.946 ppm (0.44:0.56, α-carotene: β-carotene) carotenoids and 999.485±20.023 ppm vitamin E (tocopherol and tocotrieonols), respectively and has small droplet size (119.69nm) and size distribution (0.286). No significant difference (P>0.05) was found in terms of droplet size, polydispersity index, viscosity, antioxidant activity and active contents during storage condition over the time [18]. The carotenoids and vitamin E content were within the ranges of 500–700 ppm for carotenoids and 500–1000 ppm for vitamin E in RPO as reported by previous studies [26–29]. The high carotenoids content imparts RPO with the distinct orange-red colour and its value is almost 15 times that of carrots and 300 times that of tomatoes [29]. Therefore, RPO is also one of the natural richest sources of carotenoids (pro-vitamin A). The vitamin E in RPO comes in mixed isoforms of α-, β-, γ- and δ- tocopherols and tocotrienols. Approximately 70% of the vitamin E in RPO is tocotrienols and 30% is tocopherols. Although both tocopherols and tocotrienols are sub-members of vitamin E, they have different structures. Tocopherols have long tails and lack double bonds while tocotrienols contain short tails with three double bonds at C-3’, C- 7’ and C-11’ [30]. The O/W nanoemulsion produced had spherical shape, droplet size of less than 200nm and good water solubility and antioxidant activity of 322.49±13.43 ppm compared to the control (without surfactant blend and glycerol) at 233.68±11.58 ppm. It can be concluded that the nanoemulsions act as vehicles that encapsulate lipophilic bioactive molecules and protect them from chemical degradation [31, 32] and revealed that encapsulated emulsions have higher antioxidants activity than the emulsions without emulsification.

### 3.2 Thermodynamic stability studies

The nanoemulsions were subjected to thermodynamic stability studies. Observations and results are shown in Table 1. Thermodynamic stability evaluates the physical stability of the nanoemulsions that have been subjected to centrifugation, heating-cooling cycle and freeze-thaw cycle.

**Table 1:** Thermodynamic stability studies of nanoemulsions.

Heating cooling cycle				Centrifugation		Freeze & Thaw cycle	
	Before (nm)	After (nm)	Observation	After (nm)	Observation	After (nm)	Observation
Blank	2706	553.70*	S	468.03*	S	621.83*	S, O
Full nanoemulsions	122.83	123.73	NS	116.67*	NS	129.73*	NS
Nanoemulsions without glycerol	101.43	108.23*	NS	102.67	NS	112.2*	S, O

*NS-No Separation; S-separation, O-Oiling off; * Significant difference found between before and after

Nanoemulsions that show no separation after thermodynamic stability studies indicate good and stable nanoemulsions. The result showed that the full nanoemulsions survived thermodynamic stability tests. They were stable in contrast to the control sample and the nanoemulsions without glycerol.

### 3.3 The effect of tocotrienol rich oil-in-water nanoemulsions on cell viability

The MTT assay was conducted to measure the cell metabolic activity and determine the toxicity of compounds by assessing the cellular damage. Toxicity of compounds should be screened early, as the screening helps to determine whether compounds can be further used to evaluate biology activity [33]. The viability of keratinocytes after exposure to tocotrienol-rich oil-in-water nanoemulsions (NE) after 48 hours was evaluated using MTT assay. The viability of keratinocytes was compared after treatment with different concentrations of nanoemulsions (0.35, 3.50, 8.75, 17.50 & 35.00 μg/mL), while ascorbic acid (4.4 μg/mL) was used as positive control and blank as untreated sample.

**Fig 1:**
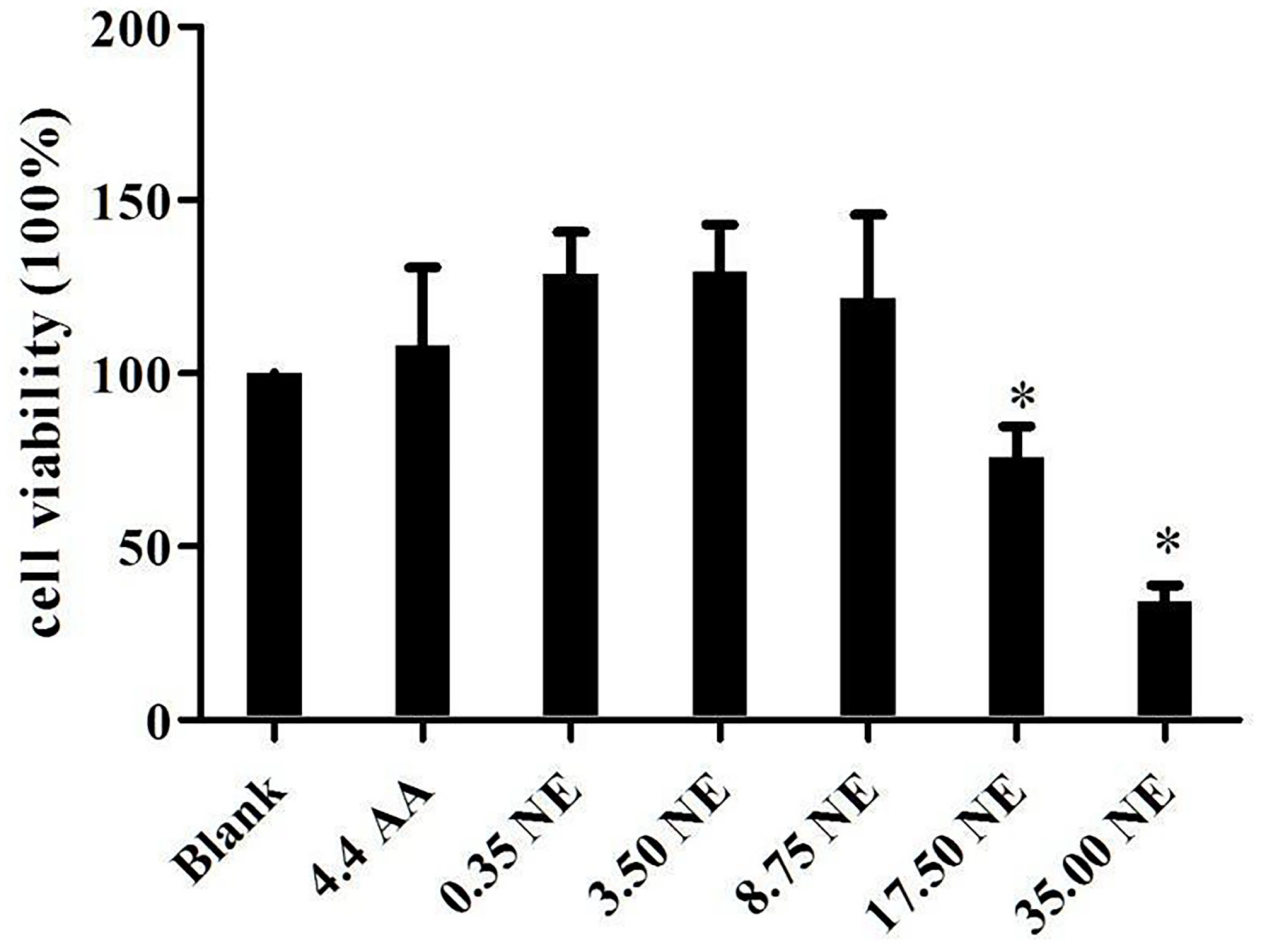
Cell viability of keratinocytes cells after different treatments. Notes: Blank = untreated samples; AA = Ascorbic acid; NE = Nanoemulsions; All concentration is in μg/mL. (*) Means significant differences found when compared to the blank.

The results showed that exposure of keratinocytes cells to NE of 0.35–8.75 μg/ml concentration resulted in an enhancement in cell viability (>100%) relative to untreated cells (Fig 1). Nanoemulsion concentrations of 0.35–8.75 μg/mL tended to have higher cell viability than blank and positive treatments but the difference among them were insignificant (P>0.05). Besides, the cell viability decreased significantly when keratinocytes cells were exposed to nanoemulsion concentrations of more than 17.50 μg/mL. This indicated that NE showed an effect on cell viability and was non-toxic to keratinocytes at concentrations less than 8.75 μg/mL, whereas keratinocytes viability decreased when nanoemulsion concentrations were higher than 17.50 μg/mL. Therefore, the dose range of 0.35–8.75 μg/mL was used for further experiments. For ascorbic acid samples, the highest concentration that can be added was 4.44 μg/ml. Higher concentrations of ascorbic acid more than was 4.44 μg/ml were toxic and effected the cell viability.

### 3.4 In-vitro assessment of nanoemulsions on wound healing effect using “scratch” model

An *in-vitro* wound healing assay based on the monolayer of keratinocytes with a “scratch” assay was performed in this study to evaluate the potential wound healing effect of tocotrienol-rich oil-in-water nanoemulsions (NE). This model is a common and advanced tool to provide an understanding and evidence on mechanisms of action and advantageous effects of actives on wound healing, especially in ethno-pharmacological research [34]. The scratch closure of keratinocytes closed significantly (P<0.05) faster in the presence of NE then nanoemulsions-free control medium (blank), where keratinocytes moved to close the wound closure by 73.76±22.18% and 67.37±4.20% at concentrations of 3.50 μg/mL and 1.75 μg/mL, respectively. The blank resulted in the slowest closure rate of 36.56±9.52% while the positive control (ascorbic acid) gave rise to 65.26±4.89% closure rate after 24 hours. These results are in agreement with a previous study done by [35], which showed that vitamin E promoted wound closure in keratinocytes. In the study, the wound closure of the keratinocytes treated with α-tocopherols was significantly faster when compared to control cells (without α-tocopherols). Interestingly, the concentration of α-tocopherols used in the previous study (4.307 μg/mL) is higher than in this present study (3.5 μg/mL, 20% of red palm oil), but the percentage of the wound closure of the previous study (~50% of control) is lower than this present study (~100% of the control). This might be due to the presence of tocotrienols in the red palm oil. It is well-known that tocotrienols is a potent antioxidant and its antioxidant potency is 60 times more effective than tocopherols [9]. This can explain why the migration of keratinocytes in this present study were more pronounced than the previous study even at lesser concentration of treatment. This was also agreed by Musalmah et al. [36], which showed that palm vitamin E was more effective in wound healing and was a better free radical scavenger than α- tocopherols. Their study assumed that a higher dosage of α-tocopherols is needed to attain equivalent wound healing effect of palm vitamin E. Another *in-vitro* experiment provided evidence that vitamin E conferred beneficial effects on wound healing through enhancing proliferation of keratinocyte. Bonferoni et al. [37] developed a nanoemulsions formulation using chitosan oleate and α-tocopherols. Their study found that treatment with α-tocopherols significantly increase the proliferation of keratinocyte at 24 hours and at seven days when compared to the control (without α-tocopherols), suggesting its potential to be used for wound and burn treatment.

**Fig 2:**
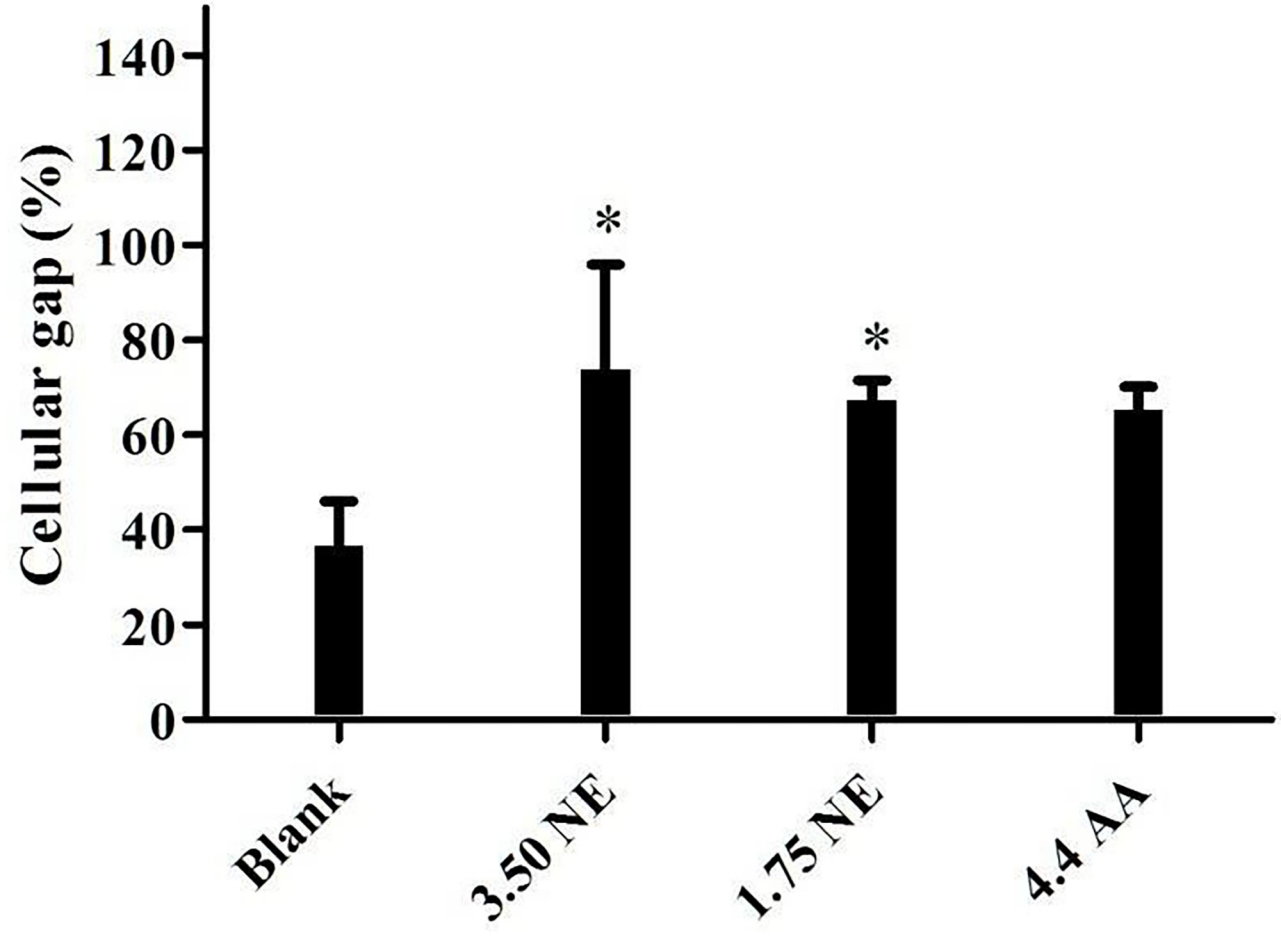
Keratinocytes cellular gap closure after different treatments. Notes: Blank = untreated samples; AA = Ascorbic acid; NE = Nanoemulsions; All concentration is in μg/mL. (*) Means significant differences found when compared to the lank.

**Fig 3:**
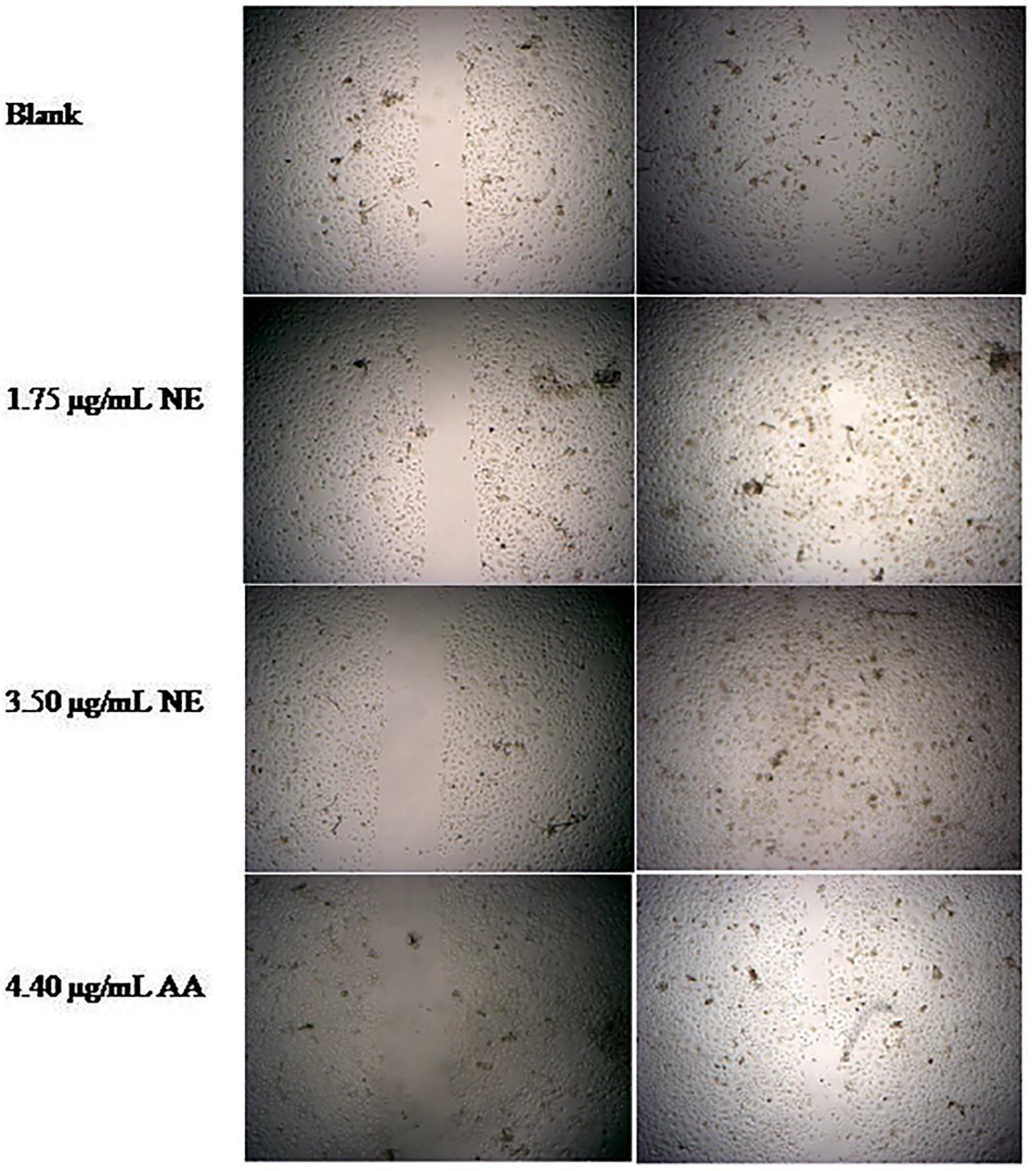
Keratinocytes cellular gap closure after different treatments. Notes: Blank = untreated samples; AA = Ascorbic acid; NE = Nanoemulsions.

Ascorbic acid, also known as vitamin C, is a strong reducing and antioxidant agent. Vitamin C is widely used for wound healing due to its antioxidant and anti-inflammatory properties which aid the recovery of wound through cell proliferation and synthesis [38–40]. The scratch closure effect of NE was higher than the positive control, however the difference was insignificant. This indicated that the wound healing effect of NE at 3.50 μg/mL was comparable to the effect of 4.4 μg/mL of ascorbic acids. The wound healing effect on scratch closure is shown in Figs 2 and 3. Overall, the highest extent of wound closure and migration of keratinocytes among different treatments was observed for the NE at 3.50 μg/mL, followed by 1.75 μg/mL of NE and 4.44 μg/mL of ascorbic acids. These results indicated that vitamin E-enriched nanoemulsion enhanced keratinocyte migration during wound healing.

### 3.5 Acute and prolonged toxicity

**Fig 4:**
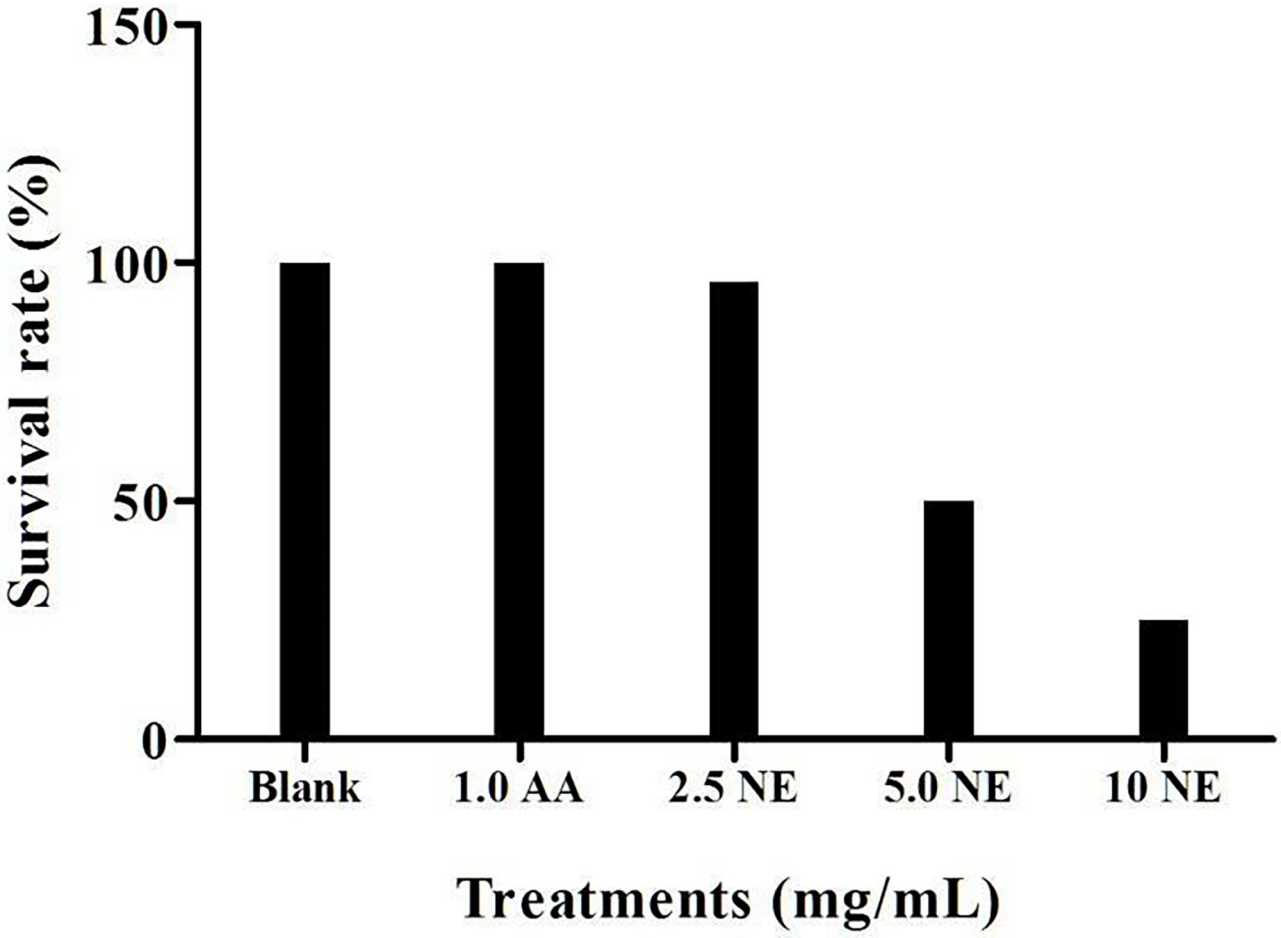
Survival rate (%) of zebrafish on nanoemulsions towards adult zebrafish at concentration of 0.25% to 1.0% (v/v) after 48 hpe (n = 10). Notes: AA = Ascorbic acid; NE = Nanoemulsions.

**Fig 5:**
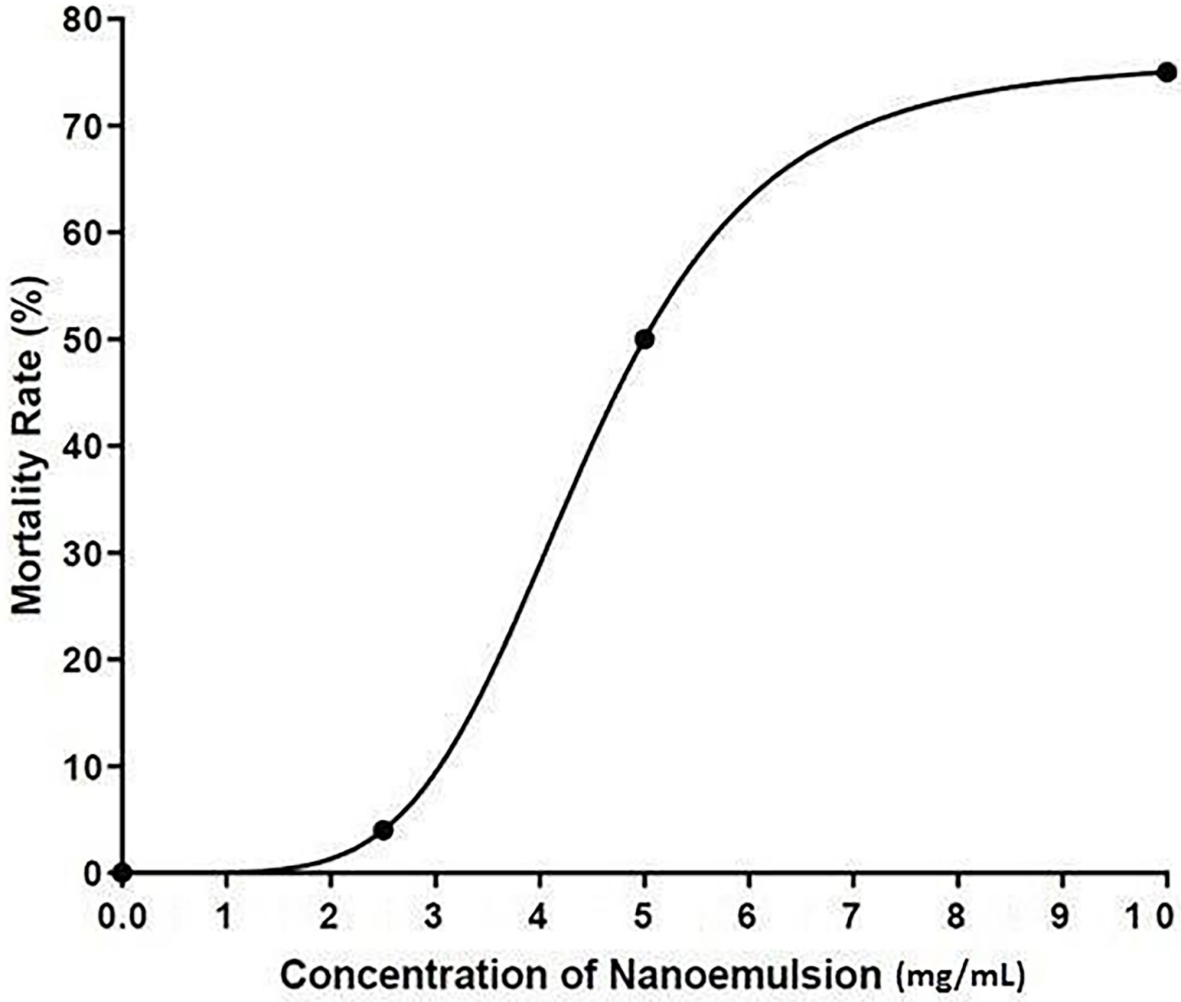
The effect of nanoemulsions on mortality rate of zebrafish (acute toxicity;n = 10).

**Fig 6:**
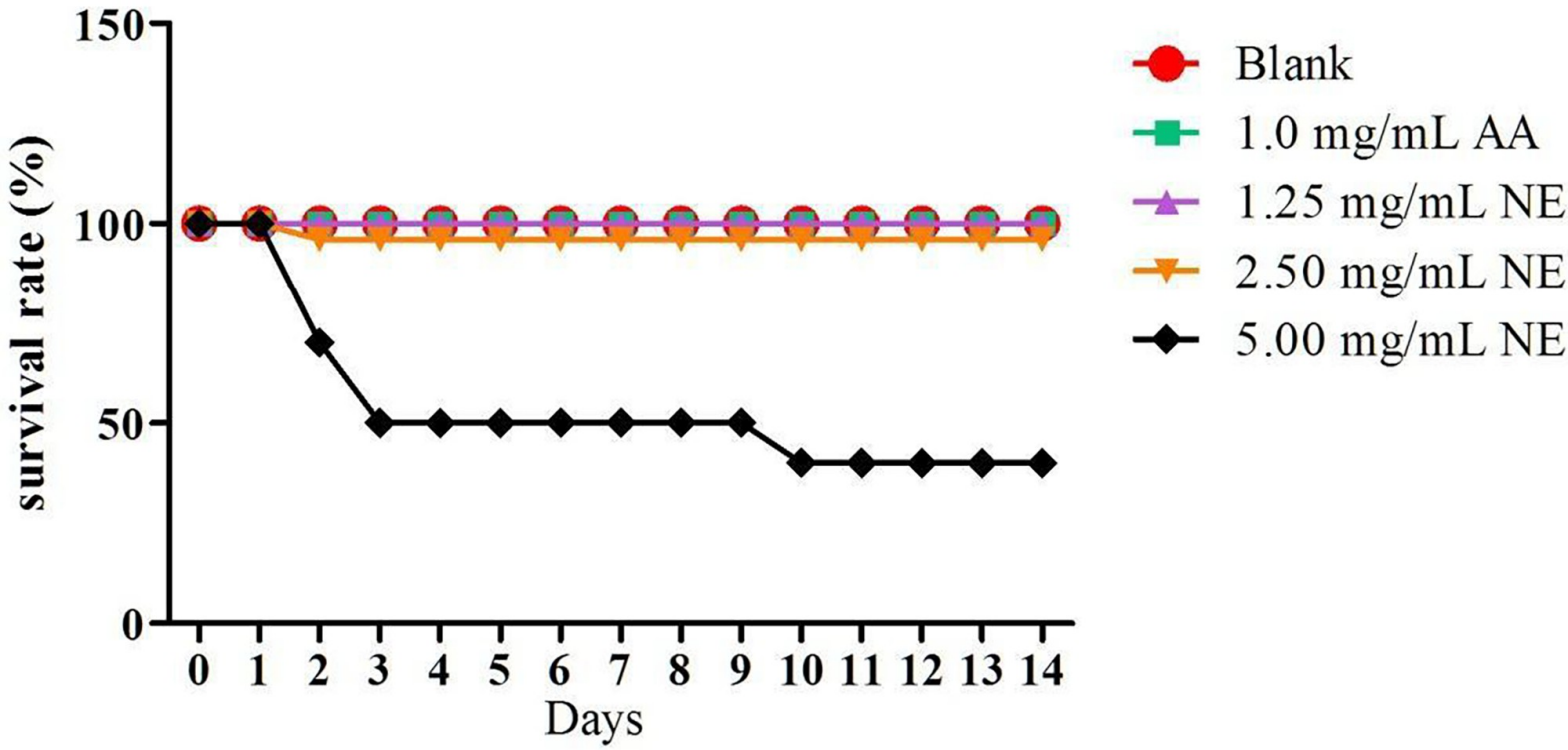
The effect of treatments on survival rate of zebrafish (prolonged toxicity;n = 10). Notes: Blank = untreated samples; AA = Ascorbic acid; NE = Nanoemulsions.

**Fig 7:**
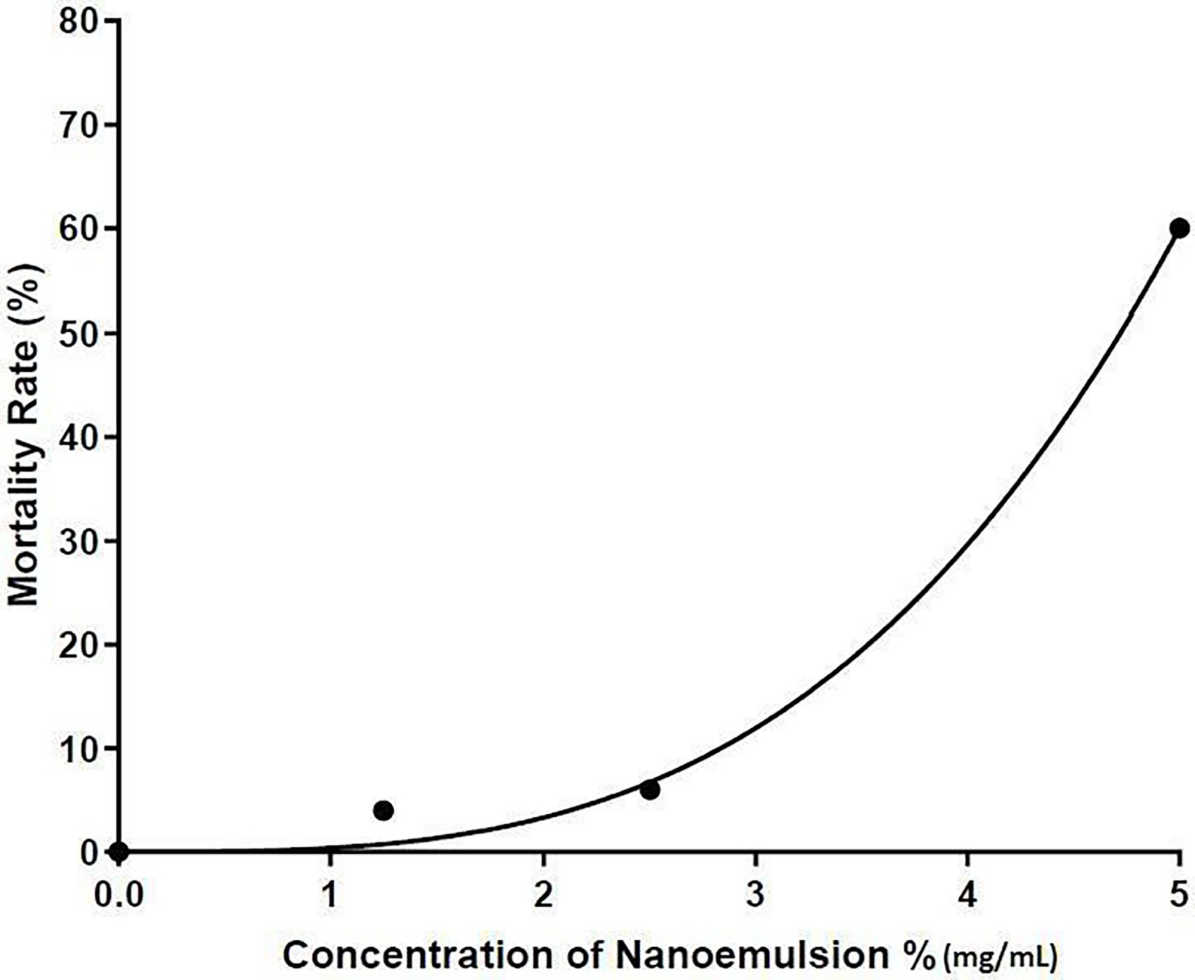
The effect of nanoemulsions on mortality rate of zebrafish (prolonged toxicity;n = 10).

Before testing the effect of wound healing on zebrafish, acute and prolonged toxicity tests were carried out to determine a concentration that is suitable to be used. Fig 4 shows the survival rate of zebrafish after 48 hours exposed to NE, blank and ascorbic acid. Concentration of ascorbic acid at 1.0 mg/mL was selected for this study as the concentration of ascorbic acid higher than 1.0 mg/mL caused death of zebrafish. The result showed that the survival rate of zebrafish decreased on exposure to high nanoemulsion concentrations, while no death was found when exposed to untreated and ascorbic acid groups. The acute lethal concentration at 50% (LC_50_ value) for adult zebrafish exposed to nanoemulsion was 5 mg/mL (Fig 5). Fig 6 shows the prolonged toxicity test (14 day post exposure, dpe) of different treatments. Results showed that exposure of zebrafish to NE less than 2.5 mg/ml showed 100% survival rate after 14 days, while 1 mg/mL of ascorbic acid and blank (untreated) share the same trend as well. The prolonged lethal concentration at 50% (LC_50_ value) for adult zebrafish exposed to nanoemulsion was 4.7 mg/mL (Fig 7). The results obtained from the acute toxicity were tallied with the prolonged toxicity results, which showed a similar LC_50_ value. From the findings, red palm oil nanoemulsions at a concentration less than 5 mg/mL can be used to test the wound healing effect in zebrafish.

### 3.6 Wound healing evaluation

Using zebrafish as a model system for studies is not a new trend, especially in the studies related to cell regeneration (wound healing, tissue regeneration, damage organs). It is worth noting that zebrafish contained around 70% genetic homologies with humans in which around 84% of the genome that causes human disease have zebrafish orthologs [41, 42]. Other than that, vertebrate such as zebrafish has a high regenerative ability. Zebrafish can restore and regenerate their lost or damaged tails, fins, heart and spinal cord morphology and functions completely, unlike mammals that experience substantial tissue loss which leads to the constitution of scar tissue, despite being able to regenerate damaged tissue.

**Fig 8:**
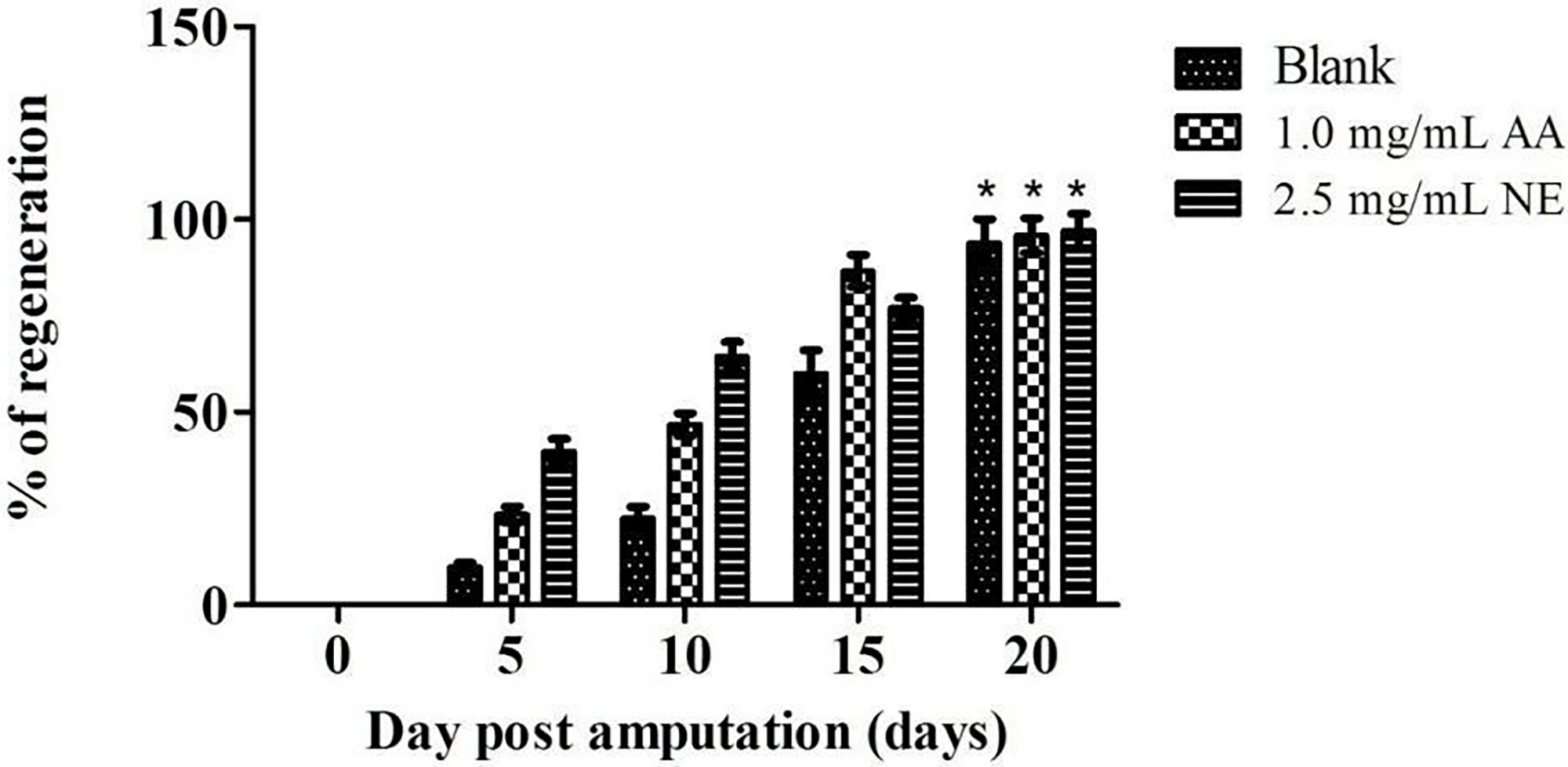
The effect of treatments on zebrafish tails regeneration (n = 10). Notes: Blank = untreated samples; AA = Ascorbic acid; NE = Nanoemulsions; * means no significant difference from each other.

**Fig 9:**
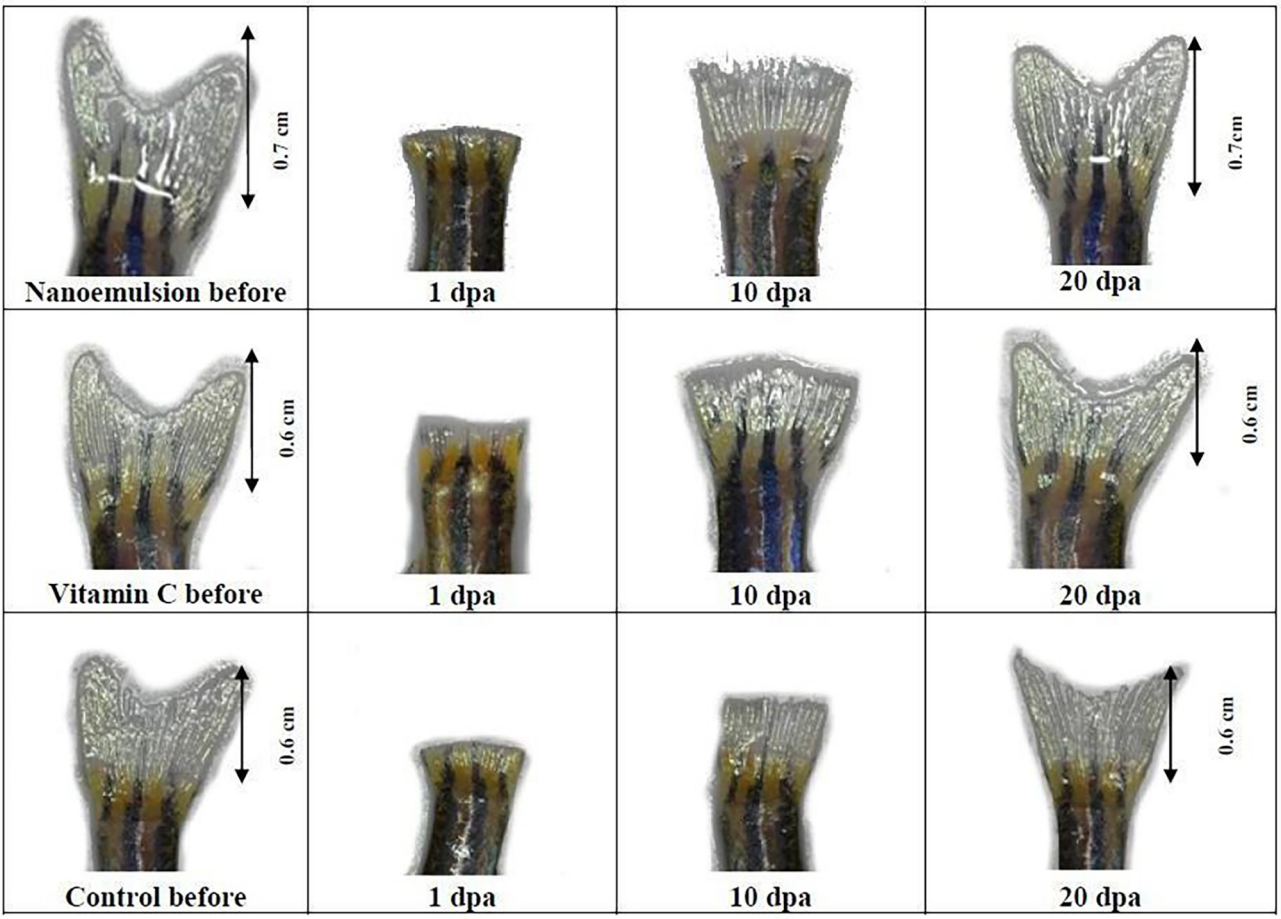
Adult zebrafish tail regeneration before and after treatments.

In the present study, the potential wound healing effect of NE was evaluated using excision zebrafish tail experiments. The zebrafish tails regeneration indicates the wound closure properties. The progress was observed and recorded from time to time. The results were plotted and compared with untreated and 1 mg/mL of ascorbic acid groups (Fig 8). Untreated zebrafish exposed to nanoemulsions-free samples exhibited slower tail regeneration growth than the zebrafish treated with NE and ascorbic acid (P<0.05). For the first ten days, NE at 2.5 mg/mL of concentration regenerated the excision zebrafish tails more efficiently than the ascorbic acid group (1 mg/mL), and then slower growth was found for the next ten days (P<0.05). The excision tails of zebrafish overall took 20 days to grow back to their original size. The improvement in tail regeneration produced by NE compared with untreated and ascorbic acid is evidently demonstrated in Fig 9.

During the first 10 days, nanoemulsions accelerated the tail regeneration rate of zebrafish by 40–60%. Tails of zebrafish treated with NE grew to 40% on the fifth day of treatment and reached more than 60% on the tenth day of treatment, which were significantly greater than the untreated and ascorbic acid treated groups (Fig 9). These results indicated that NE accelerated wound closure. The key goal for wound management is enhancing wound healing by boosting the healing process. These results showed that NE may have the potential to improve wound healing. Accelerated wound closure can greatly decrease the risks of infection, contamination and morbidity, which probably will minimize the risk of complications and mortality later.

The accelerated wound healing effect is believed to be contributed by the high content of strong antioxidants (carotenoids and vitamin E) from the red palm oil. The carotenoids and vitamin E improve wound healing in many different ways. For example, decrease inflammation, scavenge free radicals, enhance cell proliferation and improve cell remodelling in wound healing. These properties are well documented in many animal and human studies [43–49]. Although the detail and exact pathway of wound healing by vitamin E is unclear, but from previous animal and human studies done in the past, we can postulate that red palm oil may enhance the proliferation of the epithelial cells and blastema cells to produce an adequate number of cells that can replace the missing tails. During the process, the cells can be differentiated into different types of cell to resume complete morphology and function.

When compared to ascorbic acid, the tail regeneration treated with NE grew faster for the first ten days. This indicated that NE accelerated wound closure at the beginning of the healing process, which greatly reduced the risk of infection and complications later in life. Other than a strong antioxidant, vitamin E available in red palm oil, and the presence of carotenoids in red palm oil also contributed to wound recovery and the effect of vitamin A on regenerating cells is well documented. Vitamin A is essential and required for promoting organ development and maturation [50]. Previous studies reported that vitamin A was able to regenerate the damaged lung alveoli [50, 51]. The beneficial effect on cell regeneration of vitamin A was also supported by other previous studies which found that vitamin A plays an important role in regenerating animal limb. It is documented that, vitamin A has the function of re-specifying the positional memory of the limb blastema and stimulating the formation of blastema progenitor cells, then regenerate the limb of the animals [52, 53].

Besides, the concentration of nanoemulsions (2.5 mg/ml) used in the experiments was higher than the ascorbic acid (1.0 mg/ml) used and the NE consisted only 20% of red palm oil. This showed that antioxidant content in nanoemulsions is lower than the ascorbic acid. The healing potential of a lower antioxidant content in NE may be attributed to the combination of two potent antioxidant agents (vitamin E and carotenoids). It has been shown that a combination of different antioxidant agents achieved better wound healing outcome. Ehrlich et al. [54] found that animal models supplemented with a combination of vitamin E and vitamin A synthesized and yielded more cells and collagen significantly. During the anti-oxidation process, the antioxidant may be depleted rapidly and need other antioxidants to replenish the depleted antioxidant. The study revealed that carotenoids could replenish the depleted vitamin E in the process by transferring electrons to tocopheroxyl radical and regenerate tocopherols [55]. Study by Al-Kaisy & Sahib [56] reported that the combination of different vitamins achieved better performance. In their study, patients treated with topical cream that contained a combination of vitamin E and vitamin C improved burns significantly. According to the results, rapid regeneration growth of excised zebrafish tails was found in nanoemulsions group (<50%) for the first ten days while faster growth at the 15 days was found in the ascorbic acid group (~80%). Nevertheless, the wound healing effect of ascorbic acid is undeniable as it is well documented in many animals and human studies. Studies showed that vitamin C enhanced wound recovery by increasing the synthesis of collagen and proliferation of fibroblasts, as well as decreasing inflammation [57–59].

*In-vitro* and *in-vivo* evaluations are important as they are pre-clinical evaluation before testing on the human being. The enhancement of keratinocytes migration and zebrafish tail regeneration of this study provided evidence of the beneficial effect of NE on wound healing. Healing potential may be attributed to the potent antioxidant activity of vitamin E and carotenoids in red palm oil. During the wound healing process, the detection of harmful organisms will activate macrophages to produce reactive oxygen species (ROS) [60]. ROS can cause cell damage if oxidative stress and inflammatory response are not regulated [61]. Thereby, antioxidants play a vital role in regulating oxidative stress and inflammatory response to prevent excess free radical production that can lead to damage to cells, protein and DNA. Work by Posthauer et al. [62] showed that deficiency in vitamin greatly affects cell migration and proliferation, which are prominent factors of prolonged wound recovery.

It is notable that formulations need to pass through the epidermis (stratum corneum), and reach the deeper epidermis and cells in *in-vivo* evaluation, while treatment is exposed to the cells directly in *in-vitro* experiments. The cells exposure to formulations in the *in-vitro* experiments is higher than in *in-vivo* experiments [63, 64]. Therefore, the concentration used in *in-vitro* experiments is usually lower than the concentration used in *in-vivo* experiments.

The optimum wound recovery consists of well planned cellular and bio- molecular activities in various overlapping phases, such as inflammation, cell proliferation, tissue remodeling and oxidative stress [65]. Although the details and mechanisms involved in this study are not known, the *in-vivo* and *in-vitro* results provided evidence that tocotrienol-rich nanoemulsions accelerated wound closure and aid the recovery of the wound by promoting the proliferation and migration of cells, especially keratinocytes. Further investigation is needed to provide a better understanding on the mechanism of actions of tocotrienol-rich nanoemulsion on the wound healing.

## 4. Conclusion

Our results suggest that tocotrienol rich oil-in-water nanoemulsified system using red palm oil has great potential to be used for wound recovery. The findings of this study showed that NE improved wound healing better in *in-vitro* and *in-vivo* experiments compared to the blank. Enhanced keratinocytes migration of tocotrienol-rich nanoemulsion at the concentration of 3.5 μg/mL (73.76±22.18% %) was observed when compared to the blank (36.56±9.52%) in the scratch assay. *In-vivo* model of wound healing revealed that NE at 2.5 mg/mL resulted in a significantly higher (P<0.05) regeneration rate of zebrafish tail than the untreated group (the blank). These findings indicated that NE showed potential wound healing effect through promoted wound closure.

## Supporting information

S1 Fig Size distribution intensity at Day 0 of (A) full nanoemulsions (B) nanoemulsions without glycerol and (C) emulsion without glycerol and surfactant blend.
(DOCX)

S2 Fig HPLC chromatogram of full nanoemulsion at Day 0. (DOCX)
