# Supplementary material for: In-vitro and in-vivo evaluations of tocotrienol-rich nanoemulsified system on skin wound healing
Source: PLoS One. 2022 May 25;17(5):e0267381. doi: 10.1371/journal.pone.0267381 (PMC9132311; doi:10.1371/journal.pone.0267381)
Supplement: S1 Fig — Size distribution intensity at Day 0 of (A) full nanoemulsions (B) nanoemulsions without glycerol and (C) emulsion without glycerol and surfactant blend. (DOCX) [file pone.0267381.s001.docx]

(A)


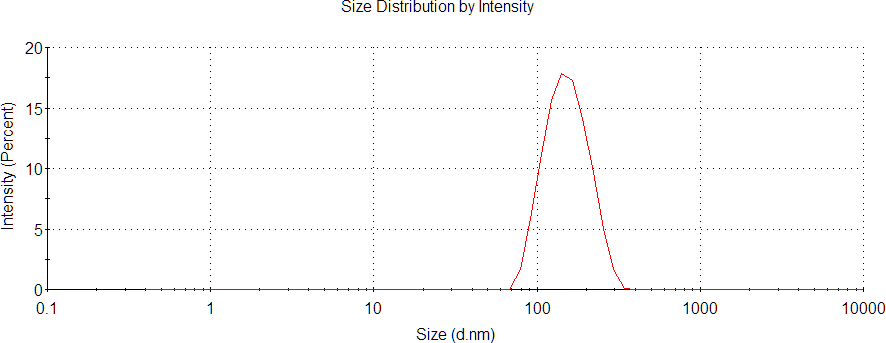


(B)


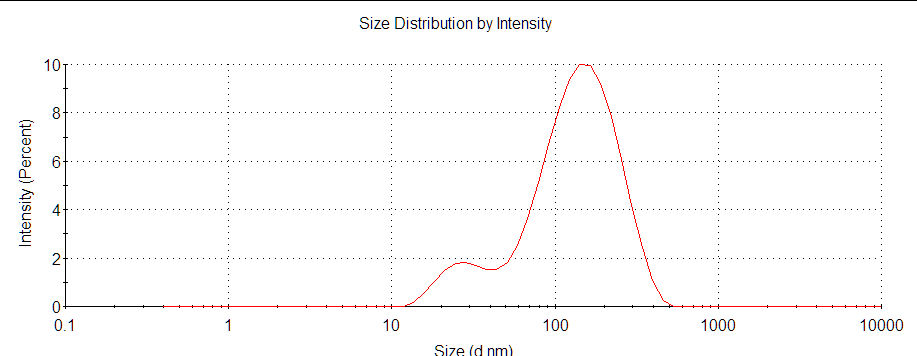


(C)


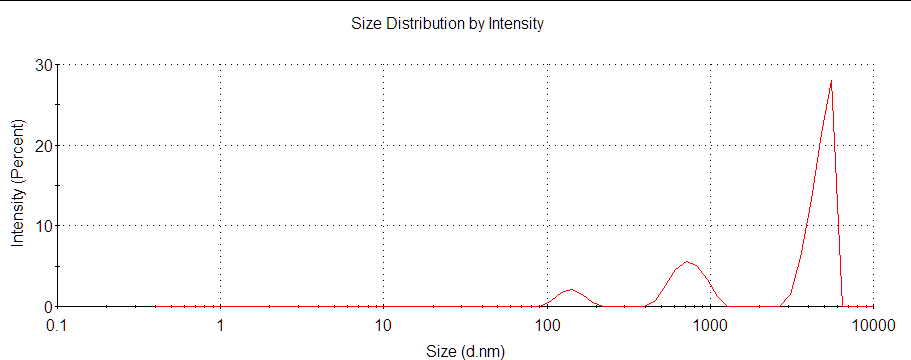


S1 Fig: Size distribution at Day 0 by intensity of (A) full nanoemulsions; (B) Nanoemulsions without glycerol and (C) emulsion without surfactant blend and glycerol.
