# Supplementary figures and images for: In-vitro and in-vivo evaluations of tocotrienol-rich nanoemulsified system on skin wound healing
Source: PLoS One. 2022 May 25;17(5):e0267381. doi: 10.1371/journal.pone.0267381 (PMC9132311; doi:10.1371/journal.pone.0267381)

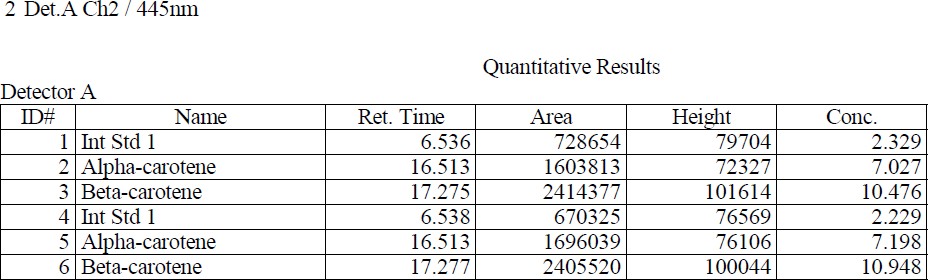

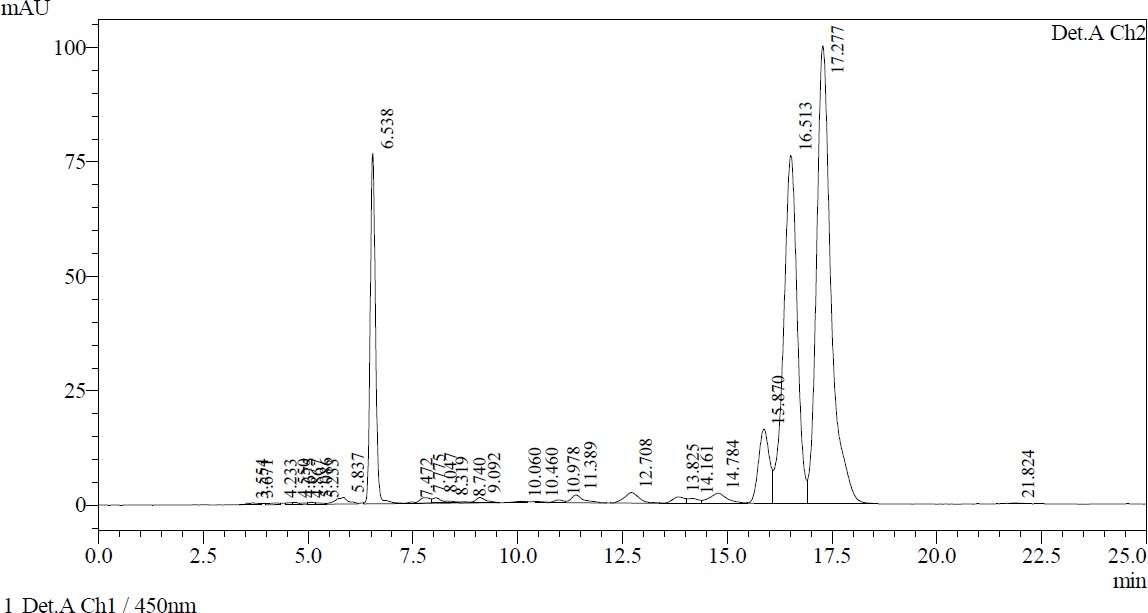

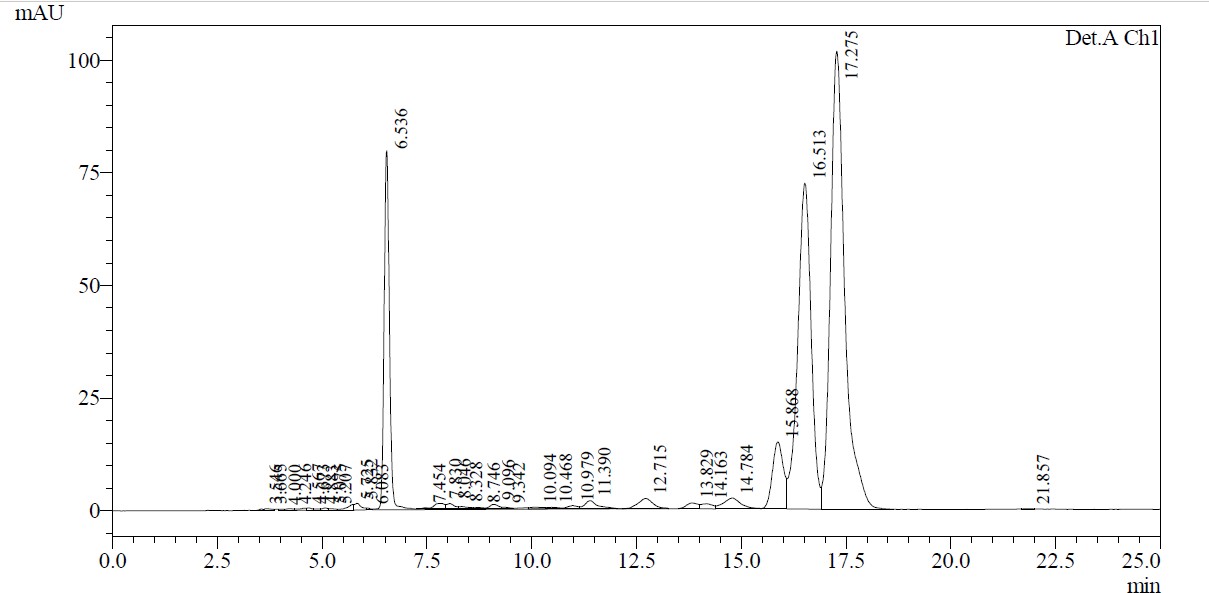


S2 Fig: HPLC Chromatograms of Full Nanoemulsions at Day 0

Supplement: S2 Fig — (DOCX) [file pone.0267381.s002.docx]
